# Self-esteem and consumption of alcohol, tobacco, and other substances in outsourced workers*

**DOI:** 10.1590/1518-8345.3401.3199

**Published:** 2019-10-14

**Authors:** Nayara Pires Nadaleti, Jefferson Felipe Ribeiro, Poliana Martins Ferreira, Sérgio Valverde Marques dos Santos, Fábio de Souza Terra

**Affiliations:** 1 Universidade Federal de Alfenas, Escola de Enfermagem, Alfenas, MG, Brasil.; 2 Bolsista da Coordenação de Aperfeiçoamento de Pessoal de Nível Superior (CAPES), Brasil.; 3 Bolsista do MEC/SESu/PET, Brasil.; 4 Universidade de São Paulo, Escola de Enfermagem de Ribeirão Preto, Centro Colaborador da OPAS/OMS para o Desenvolvimento da Pesquisa em Enfermagem, Ribeirão Preto, SP, Brasil.; 5 Bolsista da Fundação de Amparo à Pesquisa do Estado de São Paulo (FAPESP), Brasil.

**Keywords:** Occupational Health, Outsourced Services, Self Esteem, Alcohol Drinking, Tobacco Use Disorder, Street Drugs, Saúde do Trabalhador, Serviços Terceirizados, Autoestima, Consumo de Bebidas Alcoólicas, Tabagismo, Drogas Ilícitas, Salud Laboral, Servicios Externos, Autoimagen, Consumo de Bebidas Alcohólicas, Tabaquismo, Drogas Ilícitas

## Abstract

**Objective:**

to evaluate self-esteem, the consumption of alcohol, tobacco and other substances in outsourced workers of a public university.

**Method:**

a descriptive-analytical, cross-sectional, quantitative study developed with 316 outsourced workers from a municipality in the Southeast of Brazil. Data was collected through a characterization tool, the Rosenberg Self-Esteem Scale and the Alcohol, Smoking and Substance Involvement Screening Test. For data analysis, we used descriptive statistics, Pearson’s chi-square test, Fisher’s exact test, odds ratio and logistic regression.

**Results:**

the majority of workers had high self-esteem and some used alcohol, tobacco, marijuana and inhalants. A significant association between gender, age group and work shift with self-esteem; between the risk of developing problems related to alcohol consumption with sex, age group, marital status, religious belief and number of children; between the practice of physical activity and the risk of developing problems related to the consumption of tobacco products was found.

**Conclusion:**

this study contributes to the increase of knowledge due to the small number of researches involving this subject and to contribute to the nurses to have subsidies to work with this population using strategies to combat the triggers of psychic disorders.

## Abstract (pt)

**Objetivo:**

avaliar a autoestima, o consumo de álcool, de tabaco e de outras substâncias em trabalhadores terceirizados de uma universidade pública.

**Método:**

estudo descritivo-analítico, transversal, quantitativo, desenvolvido com 316 trabalhadores terceirizados de um município do Sudeste do Brasil. Os dados foram coletados por meio de um instrumento de caracterização, da Escala de Autoestima de Rosenberg e do *Alcohol, Smoking and Substance Involvement Screening Test*. Para a análise de dados, utilizaram-se a estatística descritiva, o teste de qui-quadrado de Pearson, o teste exato de Fisher, *odds ratio* e regressão logística.

**Resultados:**

a maioria dos trabalhadores possuía autoestima alta e alguns utilizavam álcool, tabaco, maconha e inalantes. Constatou-se associação significativa entre sexo, faixa etária e turno de trabalho com a autoestima; entre o risco de desenvolver problemas relacionados ao consumo de álcool com sexo, faixa etária, estado civil, crença religiosa e quantidade de filhos; entre a prática de atividade física e o risco de desenvolver problemas relacionados ao consumo de derivados do tabaco.

**Conclusão:**

este estudo contribui para o aumento do conhecimento devido ao número reduzido de pesquisas envolvendo esta temática e para os enfermeiros terem subsídios para atuação junto a esta população, utilizando-se de estratégias para combater os fatores desencadeadores de desordens psíquicas.

## Abstract (es)

**Objetivo:**

evaluar la autoestima, el consumo de alcohol, de tabaco y de otras sustancias por trabajadores tercerizados, en una universidad pública.

**Método:**

estudio descriptivo analítico, transversal y cuantitativo, desarrollado en 316 trabajadores tercerizados, en un municipio del Sureste de Brasil. Los datos fueron recogidos por medio de un instrumento de caracterización y de la Escala de Autoestima de Rosenberg y del *Alcohol, Smoking and Substance Involvement Screening Test*. Para el análisis de los datos se utilizó: estadística descriptiva, test de Chi-cuadrado de Pearson, test Exacto de Fisher, *odds ratio* y regresión logística.

**Resultados:**

la mayoría de los trabajadores poseía autoestima alta y algunos utilizaban alcohol, tabaco, marihuana e inhalantes. Se constató asociación significativa: 1) entre la autoestima con sexo, intervalo etario y turno de trabajo; 2) entre el riesgo de desarrollar problemas relacionados al consumo de alcohol con sexo, intervalo etario, estado civil, creencia religiosa y cantidad de hijos; 3) entre la práctica de actividad física con el riesgo de desarrollar problemas relacionados al consumo de derivados del tabaco.

**Conclusión:**

considerando el número reducido de investigaciones sobre esta temática, este estudio contribuye para aumentar el conocimiento y también para que los enfermeros tengan auxilio para la actuación junto a la población, utilizando estrategias para combatir los factores desencadenadores de desórdenes psíquicos.

## Introduction

In this technological, globalized and individualistic era, society has increasingly sought different forms of change to adapt to the new job configurations that are required in contracts. In this way, healthy life and social respect have become complex and secondary factors, facing the contemporary transformations and demands of the world of work, leading to outsourcing^1^.

With regard to outsourced services, this practice has become commonplace in Brazil and in the world, used as a way of increasing productivity, quality of services and reducing costs. This modality of service emerged in response to the needs of companies to seek efficient, accelerated and low-cost production and is a trend in both the public and private sectors^2-5^.

However, outsourced workers can carry out activities of low occupational qualification and low valorization, whose nature of work is manual, manual and demand physical effort. In view of this, this type of service is considered to be precarious, and may have consequences for the health and well-being of these professionals, including changes in self-esteem^5^.

In this context, it can be affirmed that the feelings of devaluation of the work, the overloads and the pressures, added to the alterations of the self-esteem, can take the worker to the consumption of alcohol, tobacco and other substances, besides aggravating preexisting diseases, such as depression and alcoholism. The use of psychoactive substances, such as alcohol, tobacco or illicit drugs, has been used as a quick and effective alternative to reduce suffering from work^6-7^.

Such substances are capable of generating greater health problems for the worker. Alcohol can cause various harm, besides leading to addiction, causing nutritional changes, cardiovascular, mental, hepatic, among others. Tobacco is among the main risk factors for the occurrence of several chronic non-communicable diseases (CNCD), such as circulatory, respiratory and cancers diseases^8^. In addition, abusive drug use entails a number of negative consequences in the person, which may include social, physical and psychological problems for the life of the individual and of society^9^.

The use of alcohol, tobacco and other substances is a public health problem that has several consequences for the health of the subject, for their work and for their interpersonal relationships, and may interfere in their quality of life^8,10^.

In view of the above and in addition to the reduced number of studies addressing such a topic related to this type of worker, the importance of investigating the self-esteem of outsourced workers and their relationship with the consumption of alcohol, tobacco and other substances is justified. This in order to subsidize information to promote the health of these individuals and, consequently, improvements in the quality of life at work.

It is inferred, considering that the nurse professional has the skills to deal with the problems that can affect the workers and to assist in their prevention, the results of the study can promote the knowledge and help this health professional to carry out preventive approaches, in a more integral and in an attempt to reduce the sickness of outsourced workers.

Thus, this study aimed to evaluate the self-esteem, consumption of alcohol, tobacco and other substances in outsourced workers of a public university.

## Method

This is a descriptive-analytical, cross-sectional, quantitative approach, developed in a public university of a municipality in the Southeast of Brazil.

The study population consisted of all outsourced workers who served this university, which accounted for 343 workers. These people worked in different areas and functions such as administrative assistant, mechanic assistant, general service assistant, lab assistant, dental assistant, among others. As there were several functions performed by these workers, and some of them with a small number of people working, it was not possible to carry out the stratification of the study population according to the variable profession. With this, it was considered all the workers classified in the outsourced category.

The criteria established for the inclusion of the participants were: to be of both sexes; be 18 years of age or older; have at least three months of service in the company; not be in varied leave such as leave health, maternity or vacations. Thus, the sample consisted of 316 workers, since 23 did not agree to participate in the study, three were on health leave and one was on maternity leave.

Data collection was performed between December 2016 and February 2017, at the workplace, without interfering with the progress of the participant’s activities. Upon being approached, the proposal of the research was presented to the worker and his voluntary collaboration and signing of the Free and Informed Consent Term (FICT) were requested and, after his consent, he was given an envelope containing the instruments used in this research. These instruments were filled by the volunteers themselves, but for those who had a low level of education or difficulty in completing, they were applied in the form of an interview, without any interference by the researchers in the interviewees’ responses.

For data collection, three instruments were used. The first one referred to a semi-structured questionnaire, with 17 questions, developed by the researchers to evaluate characterization data, life habits, chronic illness and work activities of outsourced workers, containing the following variables: gender; age; marital status; religious belief; schooling; number of children; monthly family income; type of dwelling; physical activity practice; chronic disease; use of continuous / daily medications; profession; time of operation in outsourced companies and in the current company; workload; period / shift in the current company and other employment / employment relationship.

It should be emphasized that this instrument was submitted to a refinement process, through the evaluation of five judges, and, later, a pilot test was carried out to verify its effectiveness and applicability.

The second instrument used was the Rosenberg Self-Esteem Scale to verify the self-esteem levels of the research participants. It was developed in 1965, in English, and later in 2001, translated and validated into Portuguese. It is a Likert-type scale with ten questions aimed at evaluating the positive and negative feelings of the individual, with a range from ten to 40, so the higher the score, the higher the level of self-esteem. Thus, the classification of self-esteem is obtained through the following points of cuts: score greater than 30 points = high self-esteem; score of 20 to 30 points = average self-esteem and score lower than 20 points = low self-esteem^11^.

The third instrument was the Alcohol, Smoking and Substance Involvement Screening Test (ASSIST), developed to detect the use of psychoactive substances and problems related to consumption in life and in the last three months, being translated and validated for Brazil in 2004. It has eight questions, the numbers one to seven address the use and problems related to various substances and question eight refers to the use of injectable drugs. The most used score in the studies, including this investigation, is the “Specific Substance Involvement”, which is the sum of the score for questions 2 to 7, for each substance class; therefore, the maximum possible smoking score is 31. For all other substances, the maximum possible score is 39. The interpretation of the scores refers to the risks of developing problems related to the use of substances. For alcohol, zero = no risk; from one to ten = low risk; from 11 to 26 = moderate risk; 27 or more = high risk. For the other substances: zero = no risk; one to three = low risk; four to 26 = moderate risk and 27 or more = high risk^12-13^.

The data collected was typed in an Excel spreadsheet, version 2017, for elaboration of the database; later, double typing was performed to avoid transcription errors. For the analysis of the descriptive and inferential statistics, the software Statistical Package for the Social Science, version 20.0 was used.

Pearson’s Chi-square test was used in the univariate analysis to verify the existence of an association between the self-esteem measure and the risk of developing problems related to alcohol and tobacco consumption and also to the independent characterization variables.

The level of significance was 5%, that is, the data was statistically significant for P<0.05.

It was decided to perform the tests of associations of independent variables with the risk of developing problems related to alcohol and tobacco consumption, since these two substances were the ones with the highest consumption in the studied population. In addition, the fact that the other substances presented a low frequency of consumption, made it impossible to carry out these analyzes.

Next, the odds ratio (odds ratio) was estimated using the Logistic Regression model, the Forward Stepwise selection method, the independent variables with the self-esteem measure and the risk variables to develop problems related to the consumption of alcohol consumption and the consumption of tobacco products, with a confidence interval of 95%.

Based on Resolution 466, of 2012, this study was approved by the Research Ethics Committee of the public university, according to Opinion No. 1,623,102 (CAAE: 57208316.6.0000.5142). The companies that provided outsourced services signed the authorizations to carry out the research, as well as the workers signed the FICT.

## Results

The sample consisted, mostly, of outsourced female workers (54.4%), aged between 30 and 39 years (31.3%, average 38.7 years), married or living with partners (58.2%), Catholics (70.3%), with one or two children (47.0%), monthly family income between 1,501 and 3,000 reais (51.6%, average R $ 2,642.71, corresponding to approximately 1.5 to 3.5 minimum wages), with own house (58.5%) and complete secondary education (37.0%).

It was observed that the majority of workers did not practice any physical activity (42.7%), had some chronic disease (30.1%), and systemic arterial hypertension (SAH) was the most prevalent (48.4%). It was also evidenced that, 38.6% of outsourced workers used some medication for continuous or daily use, with anti-hypertensives having a higher percentage (57.4%), followed by contraceptives (22.9%).

Regarding the labor characteristics, it was verified that part of the outsourced workers were composed of laboratory service assistants (17.7%), followed by administrative assistant, clerk and cleaning clerk (respectively, 17.4%, 16.0% and 13.0%). The majority had up to ten years of professional time in outsourced services (81.3%, average of 6.8), with a workload of 44 hours per week (84.4%, average of 43.6). (85.8%) and had another employment relationship (18.4%), with a workload of up to 20 hours per week (65.5%).

When evaluating the distribution of these people according to the self-esteem classification, it was possible to verify that 76.6% of them had high self-esteem; 22.5%, mean self-esteem and, a small part, low self-esteem (0.9%).

As a result, it was found that the substances most consumed by outsourced workers evaluated were licit (alcohol and tobacco), totaling the respective percentages of 80.1% and 43.0%, and illicit substances were marijuana and inhalants, with 10.1% and 5.1%, respectively.

Table 1 shows the distribution of outsourced workers according to the risk classification to develop substance-related problems of the ASSIST scale.

**Table 1:** Substances consumed and risk classification to develop problems in outsourced workers from public universities in Southeast Brazil, 2016/2017 (n = 316)

Substances	Risk classification	F	%
Tobacco products	Risk free	242	76.6
	Low risk	20	6.3
	Moderate risk	49	15.5
	High risk	5	1.6
		
Alcoholic beverages	Risk free	112	35.4
	Low risk	180	57.0
	Moderate risk	20	6.3
	High risk	4	1.3
		
Marijuana	Risk free	310	98.1
	Low risk	5	1.6
	Moderate risk	1	0.3
		
Cocaine / Crack	Risk free	314	99.4
	Low risk	1	0.3
	Moderate risk	1	0.3
			
Amphetamines / Ecstasy	Risk free	315	99.7
	Low risk	1	0.3
			
Inhalants	Risk free	312	98.8
	Low risk	3	0.9
	Moderate risk	1	0.3
		
Hypnotics / sedatives	Risk free	316	100.0
			
Hallucinogens	Risk free	313	99.1
	Low risk	1	0.3
	Moderate risk	2	0.6
		
Opioids	Risk free	316	10

Of all the substances mentioned in Table 1, only hypnotics / sedatives and opioids did not constitute risk factors for workers. The other substances presented some level of risk for them to develop problems associated with the consumption.

Table 2 shows the univariate analyzes of factors associated with self-esteem according to the variables that presented a significant association.

**Table 2:** Univariate analysis of the factors associated with self-esteem in outsourced workers from a public university in Southeastern Brazil, 2016/2017 (n = 316)

Variables	High self-esteem	Low / medium self-esteem	P- Value-	OR*	CI 95%^†^
Sex					
Male	119 (82.6%)	25 (17.4%)	0.020^‡^	1.000	1.101 – 3.266
Female	123 (71.5%)	49 (28.5%)		1.896	
Age group					
Up to 39 years old	127 (70.9%)	52 (29.1%)	0.007^‡^	1.000	0.267 – 0.817
More than 39 years old	115 (83.9%)	22 (16.1%)		0.467	
Work shift at the institution					
Day	202 (74.5%)	69 (25.5%)	0.035^‡^	1.000	0.139 – 0.964
Night	40 (88.9%)	5 (11.1%)		0.366	

*OR = Odds Ratio; ^†^95% CI = 95% Confidence Interval; ^‡^Pearson’s Chi-square test

In this analysis (Table 2), only the variables gender, age group and work shift in the institution had an association with self-esteem (P<0.05). Thus, outsourced workers were more likely to have low / medium self-esteem, as well as workers aged up to 39 years and those who worked during the daytime period.

The other variables, such as marital status (p = 0,171), religious belief (p = 0.774), number of children (p = 0.070), monthly family income (p = 0.871), type of housing (p = 0,370), (p = 0.377), use of continuous / daily medications (p = 0.907), time spent in outsourced services (p = 0,337), working hours in the institution (p = 0.101) ) and another employment / employment relationship (p = 0.219), did not present a significant association with self-esteem.

After analyzing the parameters of all independent variables with the self-esteem measure by the logistic regression model, it was verified that only the variables gender and age group showed statistical significance, respectively, p = 0.009 and p = 0.004, resulting in a final model adjusted. Thus, the final model found that male was a protection factor and had twice as many chances of having high self-esteem. On the other hand, workers who were aged up to 39 years were twice as likely to have low / medium self-esteem.

Table 3 presents the univariate analysis of the factors associated with the risk of developing problems related to alcohol consumption with the variables that presented a significant association.

**Table 3:** Univariate analysis of the factors associated with the risk of developing problems related to alcohol consumption in outsourced workers of public universities in the Southeast of Brazil, 2016/2017 (n = 316)

Variables	Development of problems related to alcohol consumption		P-value	OR*	CI 95%^†^

	Risk free	With risk			
Sex					
Male	42 (29.2%)	102 (70.8%)	0.033^‡^	1.000	0.375 – 0.961
Female	70 (40.7%)	102 (59.3%)		0.600	
Age group					
Up to 39 years old	54 (30.2%)	125 (69.8%)	0.025^‡^	1.000	0.369 – 0.937
More than 39 years old	58 (42.3%)	79 (57.7)		0.588	
Marital status					
With partner	77 (41.8)	107 (58.2%)	0.005^‡^	1.000	1.228 – 3.239
Without partner	35 (26.5%)	97 (73.5%)		1.994	
Religous belief					
Catholic	63 (28.4%)	159 (71.6%)	0.000^‡^	1.000	0.221 – 0.599
Others	49 (52.1%)	45 (47.9%)		0.364	
Amount of children					
No children	24 (24.7%)	73 (75.3%)	0.008^‡^	1.000	0.287 – 0.835
With children	88 (40.2%)	131 (59.8%)		0.489	

*OR = Odds Ratio; †95% CI = 95% Confidence Interval; ^‡^Pearson’s Chi-Square Test

The variables gender, age, marital status, religious beliefs and number of children had a statistical association with the risk of developing problems related to alcohol consumption (P <0.05). Thus, it was observed that being male, up to 39 years of age, not having a partner, being Catholic and having no children was more likely to develop problems associated with alcohol consumption (Table 3).

Other variables, such as monthly family income (p = 0.280), type of housing (p = 0.540), physical activity (p = 0.498), chronic disease (p = 0.933) (P = 0.979), work hours in the institution (p = 0.392), work shift in the institution (p = 0.305) and other employment/employment showed a significant association with the risk of developing problems related to alcohol consumption.

After analyzing the parameters of all independent variables with the risk of developing problems related to alcohol consumption, the logistic regression model showed that only sex, religious belief and children presented statistical significance (p = 0.034, respectively), P <0.001 and p = 0.007), resulting in a final adjusted model. Thus, males were approximately twice as likely to be at risk for alcohol-related problems as those who did not have children. In addition, respondents who were Catholics were nearly three times more likely to present such risks.

In the univariate analysis of the factors associated with the risk of developing problems related to the consumption of tobacco products, only the practice of physical activity was significantly associated with the risk of developing problems related to the consumption of tobacco derivatives (p = 0.012). Thus, those who did not practice physical activity were more likely to have this risk (OR = 0.511).

The other variables analyzed, such as gender (p = 0.733), age group (p = 0,434), marital status (p = 0.405), religious belief (p = 0.774), number of children (p = 0.837), monthly family income (p = 0.177), type of housing (p = (p = 0.668), time spent in outsourced services (p = 0.687), working hours in the institution (p = 0.364), work shift in the institution (p = 0.838), and other employment / bond (p = 0.130), were not significantly associated with the risk of developing problems associated with the consumption of tobacco products.

After the analysis of the parameters of all independent variables with the risk of developing problems related to the consumption of tobacco derivatives, the logistic regression model showed that only the practical variable of physical activity showed statistical significance (p = 0.009), resulting in an adjusted final model. In this way, people who did not practice physical activities were twice as likely to be at risk of developing problems related to the consumption of tobacco products.

Table 4 presents the analysis of the association of self-esteem with the risk of developing problems related to alcohol consumption and consumption of tobacco products in outsourced workers.

**Table 4:** Univariate analysis of self-esteem with the risk of developing problems associated with alcohol consumption and consumption of tobacco products in outsourced workers from public universities in the Southeast of Brazil, 2016/2017 (n = 316)

Variables	Development of problems		P-value	OR*	CI95%^†^

	Risk free	With risk			

	Related to alcohol consumption				
Self-esteem					
High	90 (37.2%)	152 (62.8%)	0.240^‡^	1.000	0.797 – 2.456
Average/low	22 (29.7%)	52 (70.3%)		1.400	

	**Related to the consumption of tobacco products**				

Self-esteem					
High	189 (78.1%)	53 (21.9%)	0.250^‡^	1.000	0.783 – 2.549
Average/low	53 (71.6%)	21 (28.4%)		1.413	

*OR = Odds Ratio; ^†^95% CI = 95% Confidence Interval; ^‡^Pearson’s Chi-square test

When assessing the association of the self-esteem variable with the risk of developing problems related to alcohol consumption, it was observed that there was no significant association between these two variables (p = 0.240), as well as between self-esteem and the risk of developing problems related to alcohol consumption of tobacco derivatives (p = 0.250) (Table 4).

## Discussion

It was verified, in this study, that the majority of outsourced workers have high self-esteem, while some presented medium and low self-esteem. It is well known that high levels of self-esteem may reflect adequate psychic conditions for work, however, lower levels may limit individual inspirations and achievements^14^.

This data corroborates other investigations already carried out. In a study developed with Nursing workers in the State of Minas Gerais, Brazil, it was found that 70.2% of them had high self-esteem; 29.3%, mean self-esteem and 0.5%, low self-esteem^14^. Another survey conducted in Mongolia, with workers from various companies, found that most had high self-esteem and felt confident and with a high level of engagement at work. However, workers with low self-esteem were more likely to feel emotionally destabilized and with a low engagement level^15^. Thus, with these results, it can be seen that, although the professional categories are different, the results of both investigations are similar to those of this study. In this sense, it should be mentioned that, faced with the various events of daily life, the impact of negative situations on the lives of people with high self-esteem is much lower when compared to those who have low self-esteem^15^.

It was also verified in this investigation that the substances most used by outsourced workers were licit ones, such as alcohol and tobacco. In addition, the illicit substances most used by them were marijuana and inhalants. These data are confirmed in other studies, as demonstrated below^16-17^.

In a study carried out with 403 penitentiary agents with the objective of investigating patterns of alcohol and other drug use in a state in the Northeast of Brazil, it was observed that most of them consumed alcohol, tobacco, marijuana and inhalants^16^. In a study conducted with 185 nurses from three public hospitals in a region of southern Brazil, in order to analyze the relationship between the work environment and the consumption of psychoactive substances among hospital nurses, it was found that these same substances were used at some time in their lives, with alcohol being the most consumed, followed by tobacco products, marijuana, inhalants and sedatives^17^.

In view of the above, tobacco is a strong risk factor for the development of non-communicable diseases such as cancers, Diabetes Mellitus (DM), cardiovascular diseases and lung diseases. Still, the premature deaths due to the consumption of tobacco products occur mainly in middle- and low-income countries, being a common practice among the population of workers^18^. In this way, the importance of the multidisciplinary team for the diagnosis of a smoker and the appropriate guidelines on the negative consequences of its use is realized. In addition, it is up to the nurse to perform the active search of these users, develop educational actions and minimize the risks of complications arising from this use^19^.

Concerning the consumption of alcoholic beverages, several factors occurred at work influence their consumption among workers. Among them are dissatisfaction with work, feelings of sadness at the end of the workday, and poor quality of life. This implies the consumption of alcohol by the worker, which can have consequences, such as behavioral changes, irritability, intolerance, delays in work, absences and withdrawals, and may also lead to a decrease in productivity and dismissal^20^.

Faced with this, the need for greater assistance to individuals who consume alcohol, by health teams, through immediate therapeutic interventions and, when necessary, referral to specialized services^8^.

When verifying the association of self-esteem with the independent variables, it was found that the outsourced workers were more likely to present low / medium self-esteem, as well as workers aged up to 39 years and those who worked during the daytime period.

The fact that women are more likely to develop low / medium self-esteem can be explained by their overwork. Generally, women perform both domestic duties and paid work activities, which contributes to high work hours and reduced self-esteem. Exercising long hours or having more than one employment relationship exposes women to the factors of suffering, insecurity and instability due to overwork and wear. Thus, there is a predisposition to develop low self-esteem, which can lead to physical and mental exhaustion^21^.

With respect to age, older workers have higher self-esteem, compared to younger workers, due to the fact that a higher requirement in the new generation ^22^. Moreover, it is believed that the more mature age group has a more stable self-concept and the changes faced are viewed in a positive way, besides having a greater self-knowledge, self-confidence, self-control capacity and more resilience, factors that favor high levels self-estee^23^.

Regarding the work shift, when analyzing the results of this study, it is inferred that the workers who perform their functions during the day shift have a chance of presenting changes in self-esteem due to the workload associated with the presence of another job, which can generate fatigue and lack of time to perform leisure activities. Added to these factors, excessive work hours can also lead to insecurity and high pressure at work ^21^.

It was also found in this study that being a male, up to 39 years of age, not having a partner, being Catholic and having no children are more likely to have the risk of developing problems associated with consumption of alcohol.

Regarding sex, as shown in the results presented, the highest consumption of alcoholic beverages is among men, which can be explained by the social constructions that interfere in their behaviors. The masculine characteristics are associated to the high consumption of alcoholic beverages, in the same way that this consumption is also related to the brutality of this sex, poor quality of life and health neglect when compared to women^24^.

Regarding the age group, the literature points out that young people are consuming alcoholic substances at an earlier and more intense time. The inclusion of alcoholic beverages in Brazilian society is due to the fact that these substances are legally allowed to be marketed to persons aged 18 or over, as well as the cultural and social acceptance by the population. In this context, it is emphasized that the largest consumers of alcoholic beverages are in the average age between 16 and 34 years^25-26^.

It was found in this investigation that not having a partner is a risk factor for developing problems with alcohol. There is no consensus in the literature regarding the marital status of the individual and its possible association with the consumption of alcoholic beverages. However, there are indications for a greater risk of consumption of this substance in single, divorced or widowed persons, that is, without companion^27^. Consumption of alcohol and other drugs can be an important source of marital problems, separations and divorces; therefore, there is a tendency of reduction or discontinuation of this consumption when one has companion^28^.

Regarding religious belief, the fact of being a Catholic was a risk factor for alcohol consumption. This result was different from that found in another study, in which it was evidenced that the presence of religiosity was a protective factor against alcohol consumption. Going to church or religious meetings can be seen as factors that contribute to distancing the individual from the consumption of alcoholic beverages. Experiencing a religious belief and following the precepts of a religion prove to be protective factors against alcoholism^29^.

Outsourced workers, who did not have children, were more likely to be at risk of developing problems associated with alcohol consumption, as presented previously. Having children causes profound changes in the life of the person and family, especially in their routine, since children demand care, welfare concerns and increased financial expenses. This family bond and the bond of love established with a child reduce the chances of the individual consuming alcoholic beverages^30-31^.

Based on the evaluation of the risk of developing problems related to the consumption of tobacco products, it was found that workers who did not practice physical activity were more likely to have this risk.

Smoking can influence the person’s sedentary lifestyle, being a risk factor for several incapacitating diseases, associated to the reduction of physical exercise capacity due to physical exhaustion and fatigue^32^. Physical inactivity associated with tobacco use is strongly related to overweight and obesity, increasing the risk of developing cardiovascular problems throughout life^33^. The interaction between smoking, lack of physical activity and overweight or obesity may be associated with mental health problems among young adults^34^.

In view of the above, it is important to emphasize the importance of the health team, especially nurses, in identifying the best approach to dealing with smokers, as well as their concerns, to assist them in adherence to treatment against nicotine addiction^35^.

When assessing the association of the self-esteem variable with the risk of developing problems related to alcohol consumption, it was verified that there was no significant association between these two variables, as well as between self-esteem and the risk of developing problems related to the consumption of tobacco derivatives . However, it is emphasized that self-esteem is associated with dependence on alcoholic beverages, because the greater the dependence of this substance, the lower the self-esteem of the person^36^.

Thus, it can be stated, based on the results presented, that changes in self-esteem are often related to changes in the mental health of the person associated with adherence to substance abuse such as alcohol and tobacco, in addition to non-adherence to other healthy lifestyles when compared to people with good mental health^34^.

In the context of outsourced work, it is possible to affirm that since this modality of services implies the precariousness of working conditions, workers are affected by fear of unemployment, insecurity in the work environment, psychological suffering, substance abuse, mental disorders and, consequently, incapacity for work^37^.

Therefore, prevention of mental disorders and abuses of licit and illicit substances from psychosocial factors in the work environment is paramount since mental health care will benefit not only the worker but also the people with whom he relates^37-38^.

It should be noted that the professional able to work in this area of prevention of injuries and health promotion is the nurse, since he provides extended care to the person in a critical, holistic, individual and humanized manner, considering all its dimensions^39^, including those professionals who work in companies.

Some factors were limiting in this study, such as its transversal design, which did not allow to verify the cause-effect relationship of the results found. However, it allowed to characterize and associate the variables, observing the situation of the worker at that moment. In addition, the difficulty in finding the workers was another limitation due to the different activities performed at the university. However, this factor did not interfere in the sample size and the results found. It was also added that it was not possible to carry out the stratification of the participants by profession, since there were many functions and some of them were performed by few professionals, which made it difficult to carry out the groupings and, therefore, made impossible the realization of statistical associations.

In view of the above, it is suggested to carry out longitudinal investigations that address the theme analyzed in this study in a way that demonstrates the causal and cause-effect of the change in self-esteem with the use of substances in outsourced workers.

Although this research has been conducted in a single public university, there is scope for the data to be generalized, since outsourcing is a phenomenon that exists in the current capitalist system in which there is an abrupt increase in unemployment and, along with it, growth flexibilization and precariousness of work.

This study can contribute to the advancement of scientific knowledge, since there is a reduced amount of research that addresses outsourced workers from Higher Education Institutions and the changes in self-esteem and substance use, since outsourcing is a new configuration of the labor market. Thus, the study allowed to elucidate the occurrence and influence of these factors in the work environment and may subsidize knowledge for the elaboration of actions directed to the promotion of workers’ health, improving the social, individual and labor welfare.

In view of these results, it will be possible to contribute to the fact that nurses who work in companies have subsidies for acting in their professional practice, so that they act with greater commitment in prevention, curative and educational actions to outsourced workers, greater productivity, and reduced absenteeism due to mental illness and substance use.

## Conclusion

It can be concluded that the majority of the workers had high self-esteem, and the substances most used in life were alcohol, tobacco, marijuana and inhalants. Some variables of characterization were statistically associated with the self-esteem measure and with the risk of developing problems related to the consumption of alcohol and tobacco derivatives.

In this context, it is necessary that the organs present in the outsourced companies pay attention to the factors that can cause alteration in the self-esteem and the pre-disposition for the use of substances and adopt measures that promote the quality of life at work. In addition, the importance of creating and adopting public policies that address this type of population is highlighted, with a view to promoting the health of these workers.

Thus, the presence of a nurse in companies is indispensable for the promotion of mental health and the prevention of injuries due to the work environment, since it can perform its functions using motivational strategies to combat the factors triggers of psychic disorders.
